# Complications of high volume circumcision: glans amputation in adolescents; a case report

**DOI:** 10.1186/s12894-019-0462-8

**Published:** 2019-07-11

**Authors:** Mmatsie Manentsa, Hilary Mukudu, Nthabiseng Koloane, Ashley Ringane, Eleanor Matta, Neil A. Martinson, Limakatso Lebina

**Affiliations:** Perinatal HIV Research Unit (PHRU), SA MRC Soweto Matlosana Collaborating Centre for HIV/AIDS and TB, University of the Witwatersrand, Johannesburg, South Africa

**Keywords:** MMC, Adverse events, HIV prevention, Surgical circumcision

## Abstract

**Background:**

The past four years has seen a rapid roll-out of male medical circumcision services in South Africa in response to clinical trials showing circumcision prevents HIV acquisition in heterosexual men. Clinics conduct substantial numbers of circumcisions per day. We report three cases of glans amputation in adolescents attending high volume clinics where modified Models of Optimising Volume and Efficiency (MOVE) are implemented.

**Case presentations:**

Three cases of glans amputation in young healthy men that presented for voluntary medical male circumcision. The procedures were performed by highly experienced medical officers in two cases. All these cases shared characteristics: younger males with immature genitalia, forceps guided circumcision, and likely operator fatigue. Voluntary male medical circumcision programs should include regular monitoring and evaluation and training of operators to ensure high quality surgical techniques such as working in clean areas and taking frequent breaks.

**Conclusion:**

Circumcision is a relatively simple medical procedure, however regular training and quality control in high volume Male Medical Circumcision sites is essential to prevent rare catastrophic adverse events.

## Background

In 2007 the World Health Organisation (WHO) recommended that countries with low levels of circumcision, high HIV prevalence rates and mainly heterosexual Human Immunodeficiency Virus (HIV) transmission levels should scale-up access to safe male circumcision services [1]. The WHO in its Joint Strategic Action Framework 2012–2016, outlined steps necessary to implement scale-up [2]. The framework made a call for innovative strategies to be employed in the scale-up. These strategies included innovative use of human resources through Models for Optimizing the Volume and Efficiency (MOVE) of male circumcision services [2, 3]. The MOVE model for VMMC services enhances efficiency by coordinating client flow, using multiple surgical beds to one surgical team, task shifting of some (suturing) surgical procedures to non-physicians and use of pre-packed consumables [3].

In 2010 the South African National Department of Health (DoH) began large scale roll-out of Voluntary Medical Male Circumcision (VMMC) [4]. To date approximately 2,84 million circumcisions have been conducted at multiple high-volume VMMC clinics across the country [5]. Since the roll-out began, moderate adverse events have been reported occurring in fewer than 2% of patients circumcised [5, 6]. It is important for the management of male circumcision programs both in South Africa and elsewhere that adverse events be reported as there are rare instances of severe complications, which should be monitored and responded to, thereby preventing future events [7].

Our organisation managed implementation of VMMC in five high volume clinics. Informed consent was obtained from presenting males, and for those under 18, parental consent was obtained as well. Pre-operation screening and surgical preparation was done as per the WHO’s Manual for Male Circumcision under Local Anaesthetic [6].

As part of quality control, we reviewed three glans amputations that occurred at these high volume VMMC clinics and report them as a case series. To obtain the data, a retrospective record review was conducted at the three clinics, together with one-on-one interviews conducted with medical officers and other staff members involved in the cases.

## Case presentations

### Case 1

The first case occurred in 2016 at a VMMC clinic established in 2010 in an urban township. The clinic staff numbered 26 including, medical officers, clinical associates, nurses, and support staff. On the day of the event, at total of 97 circumcisions were performed at the clinic, 22 of these by the operator involved in the glans amputation prior to the incident. The case was a 10-year-old boy who presented for medical male circumcision. The operator was a medical officer, with experience of over 13,000 circumcisions, assisted by an enrolled nurse. A total of 7 ml local anaesthetic was injected using the penile ring block technique (4,5 ml lignocaine 2% plus 2,5 ml marcaine 0,5%) [5]. As the patient’s penis was relatively small, the volume of local anaesthetic applied caused ballooning of the penis from the base to the tip of the foreskin. The forceps guided circumcision technique was used according to then standard protocols [6]. The operative procedure was initiated at 16 h10. After the operator clamped the foreskin with the forceps, the glans was palpated to ensure that it was below the clamp. When the foreskin was excised, it became clear that the glans had been amputated at a level just below the coronal sulcus.

Haemostatic control of the wound was maintained through compression and the amputated glans was placed on ice. The urologist on call was contacted to immediately assess the patient, and within 40 min the patient was taken into theatre for urgent repair and reconstruction. The glans was successfully re-attached during surgery, a catheter was inserted until the wound was healed. Based on patient feedback, he subsequently, developed a fistula and had to undergo further reconstructive surgery.

### Case 2

The second case occurred in 2013 involving a 15-year-old boy who presented for medical male circumcision at a VMMC clinic established in 2012. The clinic staff numbered 22 including managers, medical officers, nurses, and support staff. On the day of the incident 58 circumcisions had been performed, 34 of these by the operator prior to the incident. As in the previous case, after informed consent was sought from the boy's guardian, standard guidelines were followed in the screening and surgical preparation. The operator was a medical officer, who had completed 8000 circumcisions, assisted by 2 nurses. The surgical technique used in the procedure was the forceps-guided method. Local anaesthetic, 2 ml Marcaine 0,5% plus 8 ml lignocaine 2%, was injected into the base of the penis via ring block using a syringe and needle with marked swelling of the penis from the base of the penis to the foreskin. The operative procedure was initiated at 15 h19. The operator clamped the foreskin with a forceps and palpated for the position of the glans. As the foreskin was excised the operator noticed that the glans had been partially amputated and the urethra was exposed. The procedure was abandoned and haemostatic control was maintained whilst a urologist was called in to assess the patient. A partial distal glans amputation was diagnosed by the attending urologist and within 3 hrs the patient was taken to theatre for repair and reconstruction. Glans reconstruction was attempted with re-attachment of the transacted part of the glans and catheter inserted. However, post-operatively, a large portion of the reattached glans became necrotic and the patient subsequently developed a urethro-cutaneous fistula. The patient required further reconstruction.

### Case 3

The third case was a 10-year-old boy occurred at a VMMC clinic situated in a rural area. The clinic was established in 2012 and has a total staff complement of 22, including medical officers, clinical associates, nurses and other support staff. On the day of the incident, 120 circumcisions were performed at the clinic. The operator involved was a clinical associate (a cadre of staff with four years of medical training and licensed to do circumcisions) with experience of over 1000 circumcisions. He performed 25 circumcisions that day prior to the incident. The patient was consented and screened as in the previous two cases. The procedure started at 12 h30, using a modified forceps guided circumcision as the patient had phimosis. Local anaesthetic was injected by penile ring block using 3 ml lignocaine 2% and 1 ml Marcaine 0,5%. After the dorsal slit was made, preputial adhesions were released and the glans was palpated before the forceps was clamped. After the foreskin was excised the operator noted a partial amputation of the glans on the dorsal aspect, the urethra was not involved. A medical officer was immediately called to assist. A urethral catheter was inserted and haemostatic control of the wound was achieved through cauterisation and sutures. The patient was referred to a hospital for further reconstruction by a urologist. The patient has healed without sequelae.

The total number of men and boys under 15 years, and those 15 years and over circumcised in five high volume male circumcision clinics under the same administration as the three clinics where the glans amputation occurred was 17,551 and 53,782, respectively, yielding rates of glans amputation of 11.3/100000 and 1.8/100000 in those younger than 15 years, and 15 years of age or older, respectively (*p* = 0.0126).

## Discussion

Voluntary medical male circumcision is a common and safe medical procedure with frequencies of complications ranging between 1 and 15% [7]. Most adverse events are mild, but severe complications occur occasionally, and include necrosis of the glans, penile amputation, urethral fistulas and preputial fusion [7, 8]. Complications appear to be higher in older children when compared to neonatal circumcision [9]. In two cases we report, anatomy of the penis was underdeveloped. Both cases reported excessive swelling of the penis after injection of local anaesthetic making it difficult for operators to adequately palpate the glans. In such cases the forceps-guided method is not the best choice. A dissection or resection method would be a safer option.

A higher rate of adverse events have been reported with circumcisions performed by providers with less experience [9]. The operators in the first two cases were both medical officers with extensive surgical experience in MMC. However, the operator in the third case was a clinical associate who had considerably less experience. Both the VMMC clinics where these amputations occurred utilised task-shifting and task-sharing, as recommended by the MOVE principles [3, 10]. Studies have reported no difference in adverse event rates when these strategies are implemented [11]. However, the operator does not examine the patient during eligibility screening, and may only see the patient at the time of the procedure. This does not give the operator sufficient time to consider and plan alternative surgical options when presented with potentially difficult circumcisions. In the first two cases the glans amputation occurred after each operator had already completed over 20 circumcisions. All three events occurred in the afternoon when the operators were most likely fatigued.

Our experience suggests that in high volume circumcision clinics, strict rules be applied to prevent catastrophic adverse events like amputation or partial amputation of the glans. We propose that firstly, no young children (younger than 13 years) should be circumcised in the afternoon, by clinical staff that performed MMC procedures that morning. Secondly, frequent breaks (at least every 1–2 hrs) be given to operators when they doff all protective equipment, and leave the operating theatre for at least 15 min. Consideration should be given to setting a maximum daily cap on the number of men circumcised by an individual surgeon. Thirdly, in young adolescents additional care must be taken: the attending surgeon must individually examine the child before a decision to circumcise is made, thereby noting the maturity of the genitals before starting the procedure; local anaesthetic injected subcutaneously should dissipate, allowing restoration of anatomical landmarks before first incision and dissection. Fourthly, MMC guidelines should recommend the use of open surgical techniques (dorsal slit) that ensure visualisation of the glans prior to excising the foreskin and managers of VMMC programs must ensure that this is adhered to. Fifthly, there should be continuous training and retraining of all ranks of MMC providers, with on-going monitoring and evaluation to ensure quality control. Finally, implementation of a neonatal circumcision program would likely prevent such events, as a systematic literature review has shown there are minimal severe adverse events (infection, ulcers) and no glans amputations with neonatal circumcision [9].

Higher rates of adverse events have been reported during mass circumcision campaigns, mainly related to non-sterile conditions and a lack of equipment [12]. The rapid scale-up of VMMC services in South Africa has reportedly led to a decline in quality of services [10]. This decline is related to the distribution of limited resources as the programme expands and is said not to reflect the quality of surgical technique [13]. A worrying aspect of increased adverse events is the rise in medical liability litigation which may act as a barrier to the provision of VMMC [14]. Severe complications during medical male circumcision occur occasionally, and providers of VMMC services must conduct regular introspection, and quality reviews to ensure service quality is improved and adverse events that we have described, are avoided.

## Abbreviations

CDC: Centre for Disease Control
DOH: Department of Health
HIV: Human Immunodeficiency Virus
MMC: Male Medical Circumcision
MOVE: Models of Optimising Volume and Efficiency
VMMC: Voluntary Male Medical Circumcision
WHO: World Health Organisation
